# FastCellpose: A Fast and Accurate Deep-Learning Framework for Segmentation of All Glomeruli in Mouse Whole-Kidney Microscopic Optical Images

**DOI:** 10.3390/cells12232753

**Published:** 2023-11-30

**Authors:** Yutong Han, Zhan Zhang, Yafeng Li, Guoqing Fan, Mengfei Liang, Zhijie Liu, Shuo Nie, Kefu Ning, Qingming Luo, Jing Yuan

**Affiliations:** 1 Britton Chance Center for Biomedical Photonics, Wuhan National Laboratory for Optoelectronics, Huazhong University of Science and Technology, Wuhan 430074, China; hanyutong@hust.edu.cn (Y.H.); zhangzhanm@hust.edu.cn (Z.Z.); yafengli@hust.edu.cn (Y.L.); guoqingfan@hust.edu.cn (G.F.); mengfei.liang.23@ucl.ac.uk (M.L.); nieshuo@hust.edu.cn (S.N.); kfning@hust.edu.cn (K.N.); qluo@hainanu.edu.cn (Q.L.); 2 MoE Key Laboratory for Biomedical Photonics, School of Engineering Sciences, Innovation Institute, Huazhong University of Science and Technology, Wuhan 430074, China; 3 HUST-Suzhou Institute for Brainsmatics, JITRI Institute for Brainsmatics, Suzhou 215123, China; 4 School of Optical and Electronic Information, Huazhong University of Science and Technology, Wuhan 430074, China; zhijieliu@hust.edu.cn; 5 School of Biomedical Engineering, Hainan University, Haikou 570228, China

**Keywords:** whole-kidney optical imaging, deep learning, segmentation

## Abstract

Automated evaluation of all glomeruli throughout the whole kidney is essential for the comprehensive study of kidney function as well as understanding the mechanisms of kidney disease and development. The emerging large-volume microscopic optical imaging techniques allow for the acquisition of mouse whole-kidney 3D datasets at a high resolution. However, fast and accurate analysis of massive imaging data remains a challenge. Here, we propose a deep learning-based segmentation method called FastCellpose to efficiently segment all glomeruli in whole mouse kidneys. Our framework is based on Cellpose, with comprehensive optimization in network architecture and the mask reconstruction process. By means of visual and quantitative analysis, we demonstrate that FastCellpose can achieve superior segmentation performance compared to other state-of-the-art cellular segmentation methods, and the processing speed was 12-fold higher than before. Based on this high-performance framework, we quantitatively analyzed the development changes of mouse glomeruli from birth to maturity, which is promising in terms of providing new insights for research on kidney development and function.

## 1. Introduction

The glomerulus is one of the essential functional units that is widely distributed in the whole kidney. It is responsible for ultrafiltration of the blood as well as excreting waste products and maintaining fluid balance [1]. Previous studies have revealed that a series of kidney diseases, such as diabetes, unilateral ureteral obstruction, and chronic kidney disease, can lead to changes in the structure and function of the glomeruli [2,3,4,5]. Moreover, the number and size distribution of glomeruli are essential parameters for studying the mechanisms of kidney function and development [6]. Therefore, quantitative analysis of the whole-kidney glomeruli is vital for understanding the kidney in both physiological and pathological states, and has the potential for quantitative renal diagnostics.

As the centimeter-sized whole human kidney is extremely difficult to quantitatively study in 3D at the cellular level, mice are widely used as model animals for kidney research. Early studies employed histological sectioning and manual stereologic counting for the purpose of extrapolating the size and number of glomeruli in the whole kidneys of mice [7,8]. However, these techniques are essentially based on estimation rather than direct measurements, which not only is prone to errors, but also loses precise morphological information relating to individual glomeruli. To directly measure whole-kidney glomeruli, methods based on magnetic resonance imaging (MRI) have been developed [9]. However, the inherent low resolution of MRI precludes us from clearly distinguishing the structural details of glomeruli, resulting in limited accuracy of morphological analyses. To capture structural details at a higher resolution, optical microscopy is widely employed for biomedical imaging. Combined with tissue-clearing technologies, the imaging depth limitation of optical microscopies can be overcome, allowing for 3D visualization of the tissues or organs in their entirety [10,11]. A recent work demonstrated a combination of tissue clearing and light-sheet microscopy to characterize the morphology of renal glomeruli in mice [12]. However, sample deformation caused by tissue clearing may result in inaccurate measurements. Another strategy by which to explore the whole organism is the combination of optical microscopy with serial tissue sectioning. These large-volume optical imaging techniques can provide consistently high imaging quality with subcellular resolution throughout whole organs [13,14,15,16,17]. Based on this, a newly reported work has presented a cryo-fluorescence micro-optical sectioning tomography (cryo-fMOST) method to acquire complete 3D structural data of frozen organ samples with submicron resolution [18]. Using this tool, high-resolution 3D imaging data of the whole kidney with well-preserved morphology can be acquired. However, the generated massive imaging data, which contains rich structural information, poses huge challenges for subsequent auto-analysis.

A prerequisite and fundamental step for the auto-analysis of glomerular distribution is the accurate segmentation of individual glomeruli. As the acquired whole-kidney fluorescence images contain various microstructures, including the glomerular tuft, renal corpuscle, renal tubule, artery, vein, etc., it is very difficult to precisely extract the contours of the glomeruli from such complex scenes. Although many open-source algorithms and solutions [19,20,21,22,23,24] are currently available for microscope image segmentation, most of them are based on traditional image processing methods such as intensity thresholding or watershed, which cannot handle imaging data with varying signal intensities and complex structure information. Moreover, methods like CellProfiler [21,23] and ilastik [24] also rely on manual interaction and user-customized analyzing pipelines involving a series of adjustable parameters. Thus, these methods cannot be fully automated and are not applicable to the automatic analysis of large-scale 3D imaging data. In contrast, deep-learning methods can automatically and effectively handle complex imaging data. With sufficiently labeled training data, these methods can automatically extract semantic features of interested structures so as to accurately segment target structures from complex scenarios [25,26,27,28]. A series of works has demonstrated the application of deep learning in kidney segmentation using CT/MRI images [29,30] or glomeruli segmentation using whole slide images [31,32,33]. Since the deep neural network (DNN)-based methods demonstrate significantly higher accuracy, robustness, and scalability than traditional methods, they have become the most popular solution in biomedical image segmentation. Among them, U-Net [34] and Mask R-CNN [35] are the two most widely used neural networks, which have been successfully applied to various types of biomedical image data and have become the baseline for biomedical image segmentation. In 2018, Schmidt et al. developed the Stardist method for cell segmentation in microscopy images and demonstrated its superior performance on a general dataset (named Data Science Bowl) containing various fluorescence microscopy images [36]. Focusing on microscope image segmentation, Stringer et al. developed the Cellpose framework [37,38], which outperformed the established U-Net, Mask R-CNN model, and the Stardist method on various types of cells and cell-like objects. The Cellpose method can be distinguished from the existing algorithms by the introduction of a vector field output. This vector field predicted by a neural network represents the spatial gradients of the target structures, which can guide the calculation of the precise boundary of the target to be segmented. The Cellpose approach has the potential to accurately segment individual glomeruli in whole-kidney 3D datasets. However, this method does not consider computational efficiency and cannot be scaled to large-scale datasets [39]. Using this method to segment the terabyte-sized dataset would require months of time, which is unaffordable in real-world applications. Therefore, fast and accurate segmentation methods are still lacking for analyzing large-scale, high-resolution whole-kidney datasets.

In this study, we propose FastCellpose, a high-performance deep-learning framework for whole-kidney glomeruli segmentation. By pruning redundant features and simplifying the network architecture, our approach enabled a sevenfold acceleration in the network inference stage over the existing Cellpose method, while achieving higher segmentation accuracy. More importantly, we optimized the time-consuming step involved in converting the vector field output to the final binary mask and achieved 16-fold acceleration in the mask reconstruction process. Using our approach, a whole-kidney dataset can be segmented in 3.5 h with a high Dice score of 0.945, demonstrating superior performance over the existing advanced cell segmentation methods. Based on the FastCellpose framework, we segmented and analyzed the glomeruli of mouse kidneys at different ages, which, to our knowledge, is the first quantitative study on glomerular distribution changes in whole mouse kidneys during their development process. Moreover, we also demonstrated the capability of this framework in whole-brain neuron segmentation, exhibiting the potential of FastCellpose in high-throughput automatic analysis of other organs or tissues.

## 2. Materials and Methods

### 2.1. Animals

A total of 18 transgenic fluorescent mT/mG mice (Jackson Laboratory, Bar Harbor, ME, USA) of different ages (0 days, 3 days, 7 days, 2 weeks, 4 weeks, 8 weeks; three mice per age group) were used for imaging. The mT/mG mice had the expression of red fluorescence on the cell membranes of whole-body cells. All mice were fed according to the procedures and were kept on a 12 h light/12 h dark cycle. This process involving the mice was approved by the Institutional Animal Ethics Committees of Huazhong University of Science and Technology.

### 2.2. Tissue Preparation

The mice were anesthetized with a mixture of 2% chloral hydrate and 10% urethane, and subsequently transcardially perfused with 0.1 M PBS (Sigma-Aldrich, St. Louis, MO, USA) followed by 4% PFA (Sigma-Aldrich, USA). Then, the kidneys of the mice were excised and post-fixed in 4% PFA at 4 °C for 24 h. After fixation, the kidneys were rinsed overnight at 4 °C in a 0.01 M PBS solution. Next, they were dehydrated using a 30% sucrose solution until the tissue sank. Then, the kidneys were embedded using an optimal cutting temperature (OCT) compound (Sakura Finetek, Torrance, CA, USA) and frozen in liquid nitrogen immediately.

### 2.3. Cryo-fMOST for Whole-Kidney Imaging

**Figure 1:**
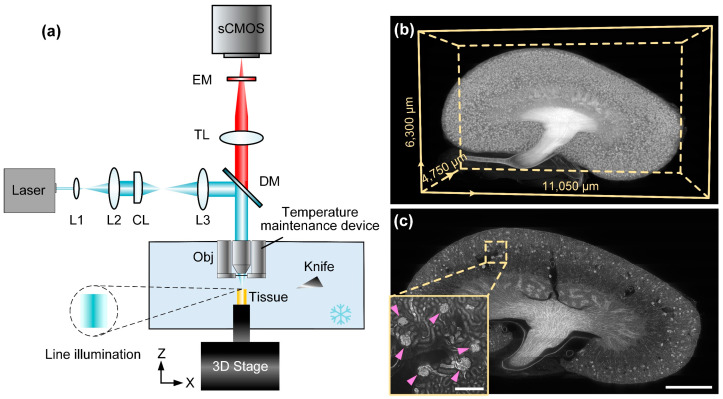
Whole-kidney imaging by Cyro-fMOST. (**a**) System configuration of Cyro-fMOST. EM, emission filter; TL, tube lens; DM, dichroic mirror; L1-L3, lens; CL, cylindrical lens; Obj, objective. (**b**) Three-dimensional visualization of the whole mouse kidney. (**c**) A typical x-y plane from the whole-kidney dataset. The enlarged area of the image, indicated by the dashed box, is shown in the bottom left corner. The pink arrows indicate the glomeruli. Scale bar, 1000 μm; 200 μm for the enlarged image.

The OCT-embedded kidneys were imaged with a Cryo-fMOST system. A brief system diagram is shown in Figure 1a. The imaging part was based on line-scanning microscopy. The laser beam from a CW diode laser (Cobolt 06-DPL 561 nm 100 mW, Hübner Photonics, Kassel, Germany) was expanded by a 4f system. The system consisted of two lenses (L1, AC050-008-A-ML, f = 7.5 mm and L2, AC254-250-A, f = 250 mm, Thorlabs, Newton, NJ, USA). The line illumination was formed by a cylindrical lens (CL, ACY254-100-A, f = 100 mm, Thorlabs, USA) and then transmitted through a lens (L3, AC254-125-A, f = 125 mm, Thorlabs, USA). The excitation beam was reflected on a dichroic mirror (DM, FF493/574-Di01–25×36, Semrock, Rochester, NY, USA) and focused on a target surface through an objective lens (LUCPLFLN 20×, NA = 0.45, Olympus, Tokyo, Japan). The excited fluorescence was filtered by DM and an emission filter (EM, FF01-620/52, Semrock Inc., USA), focused by a tube lens (TL, U-TLU, f = 180 mm, Olympus, Japan), and detected by a scientific complementary metal oxide semiconductor (sCMOS) camera (ORCA-Flash 4.0, Hamamatsu Photonics K.K., Hamamatsu, Japan). The x-y planes of the sample were obtained through line scanning, where the illumination line remained stationary and the sample was scanned by a 3D translation stage (X: XML210, Y: XMS100, Z: GTS30V, Newport, Irvine, CA, USA). After imaging, the translation stage moved the sample to the microtome (Diatome AG, Port, Switzerland) for removal of the imaged tissue. The process of imaging and sectioning was repeated until the entire kidney dataset had been acquired.

The whole kidney was imaged at a voxel size of 0.32 × 0.32 × 3 μm^3^, and the raw data volume for an 8-week-old mouse kidney had a size of 33,996 × 19,520 × 1584 voxels, corresponding to a data size of 1.5 TB. Figure 1b demonstrates the imaging results of an 8-week-old mouse kidney. It can be seen from the 3D reconstruction result that the overall morphological structure of the kidney was complete. Furthermore, the anatomical structure of the individual glomeruli can be clearly observed, as shown in the enlarged image from the x-y plane (Figure 1c). The high image quality ensures the feasibility of our subsequent automatic analysis of whole-kidney glomeruli.

### 2.4. FastCellpose Framework

The FastCellpose framework was proposed for rapid and accurate image segmentation in large-scale data. Similarly to Cellpose, it utilizes a two-step process. In the first step, the neural network learns to transform the raw input images into vector fields that represent the spatial gradients of the target structures. In the second step, it uses gradient tracking to convert the vector field output into the final mask. Compared to the original Cellpose method, the FastCellpose framework is optimized in terms of network structure and the mask reconstruction process, which significantly improves the overall processing efficiency and enables its application to large-scale data.

**Figure 2:**
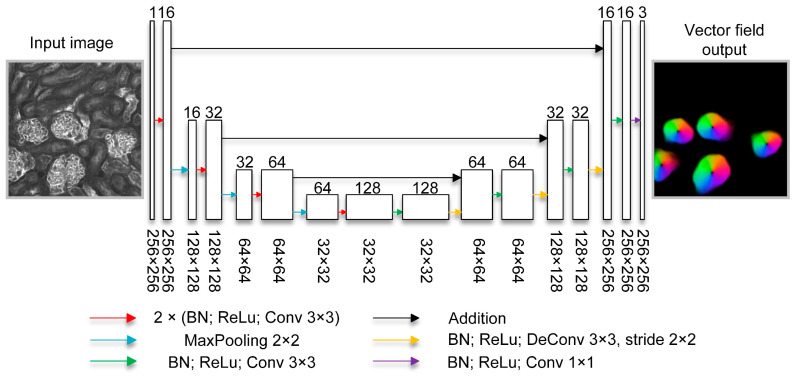
The architecture of the proposed lightweight network in FastCellpose. The number of output feature maps is denoted on the top of the block, and the numbers on the bottom of the block represent the size of the feature map. The operations are represented by arrows of different colors.

As shown in Figure 2, a lightweight U-shaped neural network was proposed for efficient gradient prediction in FastCellpose. The network consists of an encoder path and a decoder path. The encoder path is responsible for extracting abstract representations of the input data. This is achieved by stacking a series of encoding units. Each encoding unit contains two convolution layers, and each convolution layer is accompanied by a batch normalization (BN) layer [40] and a rectified linear unit (ReLU) layer. After two convolution operations, the output data goes through a max pooling layer in order to halve the image size. The output feature size for the first encoding unit (i.e., base feature size) is 16, and the subsequent encoding units steadily double the output feature size. The decoder path is responsible for recovering the hierarchy features extracted from the encoder path into the gradient vector field of the input image. Symmetric with the encoder path, a series of decoding units lie in the decoder path to gradually upsample the image to its original size. Each decoding unit contains a deconvolution and a convolution layer.

The number of encoding and decoding units is 3, and there are skip connections between layers of the same size in the encoder and decoder paths. Instead of using feature concatenation as the skip connection, additive identity mapping is employed, which is more computationally efficient. Both the input and output image sizes are 256 × 256. The input channel is 1, and the output channel is 3 (horizontal, vertical gradients, and a probability map indicating whether a pixel belongs to the interested structure).

**Figure 3:**
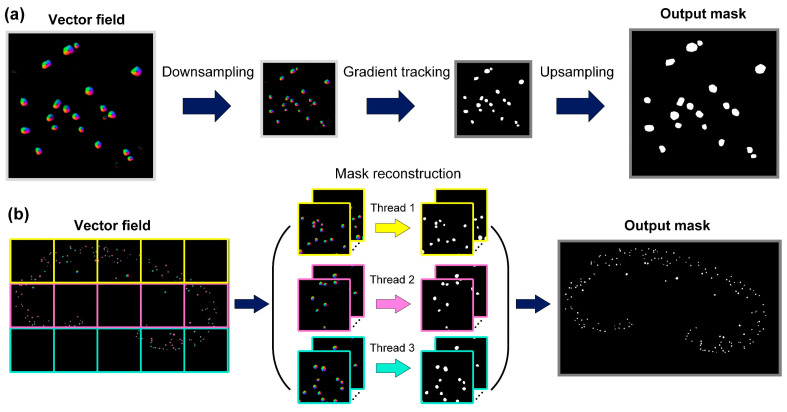
The optimized pipeline for mask reconstruction in FastCellpose. (**a**) We accelerated the gradient tracking process by downsampling the vector field and then upsampling the reconstructed mask. (**b**) We employed parallel computing to further accelerate the mask reconstruction process for whole x-y plane images.

An optimized post-processing pipeline was proposed for mask restoration from the network-predicted vector fields. As shown in Figure 3a, inspired by the downsample–upsample structure in Unet, we chose to downsample the vector field output from the network so that the gradient tracking was performed on the downsampled image, which greatly reduced the computational burden. Then, an interpolation algorithm was used to restore the output mask to its original size. This method effectively accelerated the processing speed and showed comparable accuracy to the previous method. The downsampling scale factor was set to 2 for all experiments. Moreover, we optimized the hyperparameters relating to gradient flow tracking. According to the size and shape of glomeruli, a relatively low iteration number is enough for a given pixel to converge to the center point. Therefore, instead of using an iteration number of 200, as suggested in the Cellpose article, we set the iteration number to 50 in our FastCellpose framework. Furthermore, we employed parallel computing to further accelerate the reconstruction process, as shown in Figure 3b. For a whole x-y plane image, we first cropped it into a series of sub-images of 2048 × 2048 pixels. Then, multiple threads were assigned to simultaneously perform the gradient flow tracking process. In this way, the processing throughput of the computing unit was fully utilized, resulting in higher computational efficiency. Finally, the segmented x-y plane image was obtained by fusing all the reconstructed sub-images in order.

To summarize, the difference between the original Cellpose method and the FastCellpose method lies in the network architecture and the mask reconstruction process. Instead of using a deep and large network, as in the Cellpose method, the FastCellpose method employs an optimized lightweight network for fast and efficient computing. Furthermore, compared to the Cellpose method, the FastCellpose method showed 3 improvements in the mask reconstruction component, including performing gradient tracking in downsampled vector fields, reducing the iteration number, and employing parallel computing. These optimizations aim to improve the segmentation performance and efficiency.

### 2.5. Training Data Preparation and Implementation Details

To obtain training data for the base FastCellpose model, we randomly selected 10 x-y plane images at an axial interval of 330 μm from an 8-week-old mouse kidney dataset. The ground-truth (GT) masks of the above images were obtained by experts’ manual annotation. The ground-truth spatial gradient maps were generated through simulated diffusion on the mask images. Afterwards, the ground-truth images along with the raw input images were cropped into 3000 paired image patches with image sizes of 256 × 256 pixels. The training set was generated by randomly selecting 2500 image pairs from the above data. The remaining 500 image pairs served as the validation set. The test set contained 10 raw and ground-truth mask images with pixel sizes of 2048 × 2048 that were randomly cropped in the 8-week-old whole-kidney dataset and not included in training or validation sets. The loss function for the FastCellpose model is defined as:

(1) $$\left\{ \begin{array}{l} L_{total}=L_{gradient}+L_{prob}\\ L_{gradient}={\left\|C_{0}-\lambda H\right\|}_{2}+{\left\|C_{1}-\lambda V\right\|}_{2}\\ L_{prob}=-(1-P)\log\left[1-\sigma (C_{2})\right]-P\log\left[\sigma (C_{2})\right] \end{array}\right.$$

where *C_i_* is the *i*-th channel of the network output, and *H* and *V* denote the horizontal and vertical ground-truth gradient maps, respectively. *λ* is a weighted parameter for balancing the contribution of the gradient loss and probability loss, and is set as 5. *P* is the ground-truth mask, and σ denotes the sigmoid function.

The network parameters were optimized via an SGD optimizer with a momentum of 0.9 and a weight decay of 0.00001. The batch size was set as 4, and the network was trained for 150 epochs. For the first 10 epochs, the learning rate started at zero and linearly increased to 0.2. For the next 90 epochs, the learning rate was fixed at 0.2. And for the final 50 epochs, the learning rate decayed by half after every 10 epochs. We saved the network snapshot after each training epoch and evaluated its performance on the validation set. The best model was found with the highest segmentation performance on the validation set. The network model was constructed using the Pytorch framework (version 2.0.1). Training and testing of the FastCellpose framework was performed on a workstation equipped with a Nvidia GeForce RTX 3090 GPU card, an Intel(R) Xeon(R) Gold 5222 CPU, and 128 GB of RAM.

### 2.6. Performance Criteria

The segmentation performance of our framework was evaluated using two commonly used criteria, i.e., *Dice* and Intersection over Union (*IoU*), which are defined as:

(2) $$\left\{ \begin{array}{l} Dice(P,G)=\frac{2\left|P\cap G\right|}{\left|P\right|+\left|G\right|}\\ IoU(P,G)=\frac{\left|P\cap G\right|}{\left|P\cup G\right|} \end{array}\right.$$

where *P* and *G* denote the algorithm-segmented image and the ground-truth mask image, respectively. Both the *Dice* and *IoU* were between 0 and 1, with a higher score indicating a better segmentation performance.

## 3. Results

### 3.1. Evaluation of Segmentation Algorithms for Glomeruli Segmentation

**Figure 4:**
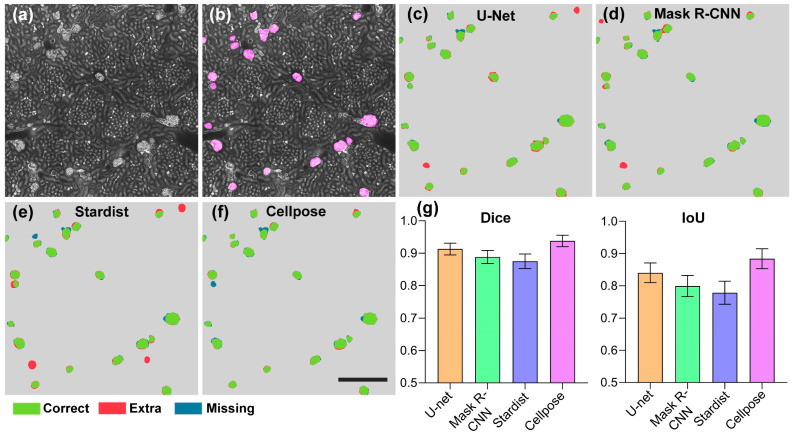
Quantitative comparison of advanced deep-learning-based segmentation methods on glomeruli segmentation. (**a**) A typical image from the whole-kidney dataset. (**b**) The GT masks of glomeruli (pink) are shown overlaid with the original image. (**c**–**f**) Segmentation results of different methods on the image shown in (**a**). Scale bar, 400 μm. (**g**) The Dice and IoU scores of the U-Net, Mask R-CNN, Stardist, and Cellpose methods. Correctly segmented glomeruli (true positive) are colored in green. Extra (false positive) and missing glomeruli (false negative) are in colored red and blue, respectively.

Toward the goal of developing high-performance automated algorithms for whole-kidney glomeruli segmentation, we first searched for well-performing algorithms for further optimization. We quantitatively evaluated and compared the current state-of-the-art DNN-based segmentation algorithms on the test set. In Figure 4a,b, a typical test image and the corresponding ground-truth label are demonstrated.

We selected four representative microscope cell segmentation algorithms, including U-Net, Mask R-CNN, Stardist, and Cellpose, for a comparison of their performances. For an intuitive comparison of the segmentation results, we demonstrated the visualization results as shown in Figure 4c–f. It can be clearly seen that in the segmentation results of U-Net, Mask R-CNN, and Stardist, wrongly identified and missed glomeruli were common. In contrast, the Cellpose algorithm accurately identified the vast majority of glomeruli, and the output contour was close to GT. We also demonstrated the quantitative statistical results, as shown in Figure 4g. The Dice and IoU values of the Stardist algorithm on the test set were 0.875 ± 0.022 and 0.778 ± 0.036, respectively. The Mask R-CNN model slightly outperformed Stardist and obtained Dice and IoU values of 0.888 ± 0.020 and 0.799 ± 0.033, respectively. The U-Net model outperformed the Stardist and Mask R-CNN algorithms on the kidney dataset and had Dice and IoU values of 0.913 ± 0.018 and 0.840 ± 0.031, respectively. Compared with the above methods, the Cellpose model, which employs spatial gradients as intermediate representations, achieved a significantly higher segmentation performance, with Dice and IoU values of 0.938 ± 0.018 and 0.884 ± 0.031, respectively. These results indicate that Cellpose showed a superior performance in kidney glomeruli segmentation. However, the high segmentation performance came at the cost of an extremely low reconstruction time. Even for a high-performance workstation, the segmenting time of Cellpose for an image with a 2048 × 2048 pixel size reached 6.18 s, which is unaffordable in the application of large-scale data. Therefore, optimization of the reconstruction efficiency of Cellpose is direly needed.

### 3.2. Comprehensive Optimization of FastCellpose for Rapid Image Segmentation

To accelerate the processing speed of the original Cellpose framework, we first optimized the network architecture and network deployment. Since a large network was employed in the Cellpose framework, a series of issues including long training and inference time, large memory consumption, and overfitting would arise when directly applying it to specific tasks. Therefore, we made the corresponding simplifications. Specifically, we removed the style branches, as they were designed for simultaneously segmenting different types of cellular images. Moreover, we modified the upsampling module. We employed the deconvolution layer to replace the original interpolation-based upsampling layer and a subsequent convolution layer, making the network more compact. More importantly, we optimized the network width and depth parameters, as they are key parameters that impact the overall performance of the network. In the U-Net-based network, the width and depth of the network are determined by the number of base feature maps (denoted as K) and the number of convolutional layers in each encoding/decoding unit (denoted as N), respectively. Therefore, we investigated the comprehensive performance of the network under different combinations of the K and N parameters. As shown in Table 1, the best model was found with K and N values of 16 and 2, respectively.

**Figure 5:**
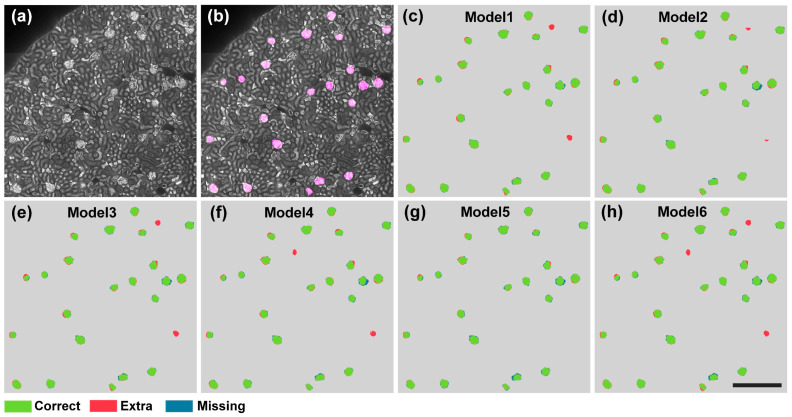
Segmentation results of different models. (**a**) A typical image from the whole-kidney dataset. (**b**) The GT masks of glomeruli (pink) are shown overlaid with the original image. (**c**–**h**) Segmentation results of different models on the image shown in (**a**). Scale bar, 400 μm. Correctly segmented glomeruli (true positive) are colored in green. Extra (false positive) and missing glomeruli (false negative) are colored in blue and red, respectively.

Compared with the original Cellpose model, our lightweight network compressed the network parameters by 92%, thus reducing the inference time and memory consumption by 67% and 78%, respectively. Moreover, as the overfitting problem was effectively suppressed, our network achieved an even higher segmentation performance than the original large model (Figure 5). The Dice and IoU scores were 0.947 ± 0.017 and 0.899 ± 0.031, respectively. Furthermore, we implemented our lightweight model using TensorRT (Nvidia). This optimization in hardware deployment further accelerated the network inference time by 2.3 times, resulting in an overall sevenfold speed improvement in the network inference stage.

Another time-consuming step in the original Cellpose framework is the mask reconstruction process, which accounts for 75% of the entire reconstruction time. To accelerate this process, we proposed a novel strategy that performed gradient tracking on the downsampled network output image, and then upsampled the restored mask image to obtain the final segmentation result. This was based on our finding that reducing the sizes of the gradient maps within a certain range has a negligible impact on the results of mask reconstruction, as long as the network can accurately predict the horizontal and vertical gradients. Using this strategy, the heavy computational burden related to the gradient tracking process can be reduced effectively, leading to a higher processing speed. After systematic comparison, the downsampling scale factor was set as 2 (Table 2).

We demonstrated that the segmentation results obtained using this strategy achieved similarly high Dice and IoU values (0.945 ± 0.016 and 0.894 ± 0.028) to the previous ones, while the reconstruction speed was 3.5-fold higher than before. We also demonstrated that if segmentation were to be performed directly on the downsampled original image, the accuracy would be significantly reduced, with Dice and IoU values of only 0.810 ± 0.050 and 0.683 ± 0.068, respectively. This result demonstrated the superiority of our method. We further optimized the reconstruction pipeline by combining hyperparameter optimization and parallel computing, achieving a total 16-fold acceleration in the mask reconstruction process. Using the FastCellpose framework, the network inference time and mask reconstruction time for an image with a 2048 × 2048 pixel size were only 0.22 s and 0.28 s, respectively. The overall segmentation speed was 12 times faster than before. We demonstrated a comparison of the performance of the FastCellpose method with other advanced cellular segmentation methods, as shown in Table 3. The results show that our method achieved the state-of-the-art level in either segmentation accuracy or efficiency.

**Figure 6:**
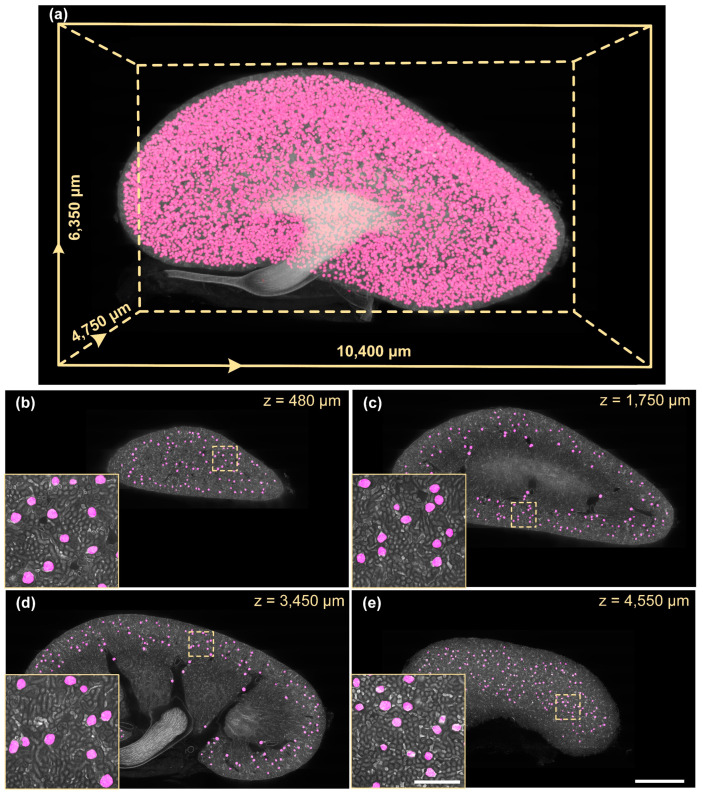
Whole-kidney glomeruli segmentation results using FastCellpose. (**a**) Three-dimensional visualization of the whole-kidney glomeruli segmentation result. (**b**–**e**) Four coronal sections at different axial positions are demonstrated. Segmentation results (magenta) are shown overlaid with the original data. Scale bar, 1000 μm; 200 μm for the enlarged image.

We segmented an 8-week-old mouse whole-kidney dataset using FastCellpose, as shown in Figure 6.

**Figure 7:**
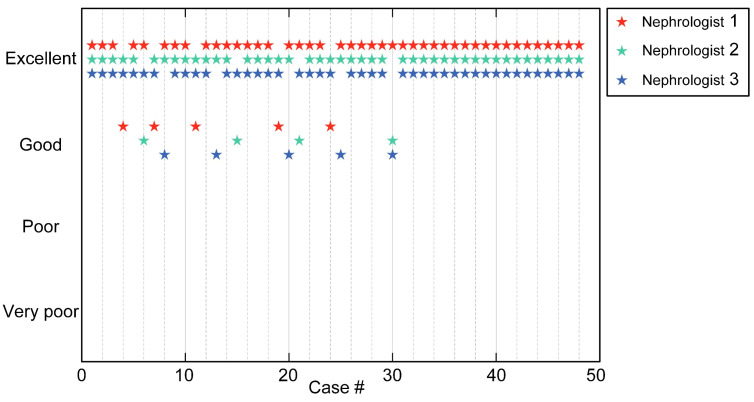
Qualitative evaluation by three nephrologists to validate the segmentation quality generated by FastCellpose.

The 3D visualization of the whole-kidney glomeruli segmentation result is demonstrated in Figure 6a. We further selected four different coronal sections from the segmented results for visual inspection (Figure 6b–e). It can be seen that, although the structure and morphology of the kidney manifested significant differences at different coronal planes, the FastCellpose model was able to accurately identify all the glomeruli in the image, and the segmentation contour matched well with the edges of the glomeruli. To further validate the accuracy of FastCellpose in whole-kidney glomeruli segmentation, we performed a study involving professional nephrologists. In this blind study, we randomly selected 48 coronal images with the corresponding segmentation results from the mouse whole-kidney dataset to generate a test set. Three nephrologists independently evaluated the segmentation results generated by FastCellpose and rated the quality (Figure 7). The statistical results showed that all images had good segmentation quality, and over 90% of the segmentation results were considered excellent. These results demonstrate that our FastCellpose framework has the capability to automatically and accurately segment all glomeruli in an entire kidney.

We also counted the time consumption needed to segment the whole dataset on a normal workstation. It took only 3.5 h for FastCellpose to segment a whole-kidney dataset with 1.3 × 1.3 × 3 μm^3^ voxel resolution, demonstrating the high efficiency of the proposed FastCellpose framework.

### 3.3. Quantitative Analysis of Glomerular Development

**Figure 8:**
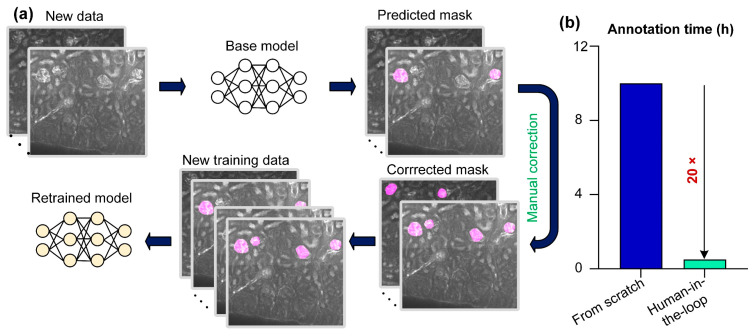
A human-in-the-loop approach for applying FastCellpose to accurately segment the kidney datasets of different ages. (**a**) Schematic of the human-in-the-loop approach. (**b**) Annotation time of the traditional (manual annotating from scratch) and the human-in-the-loop approach.

As there are significant differences in the morphology and structure of the kidneys of mice of different ages, the base FastCellpose model obtained by using the training data from an 8-week-old mouse kidney would not perform equally well on imaging data of other mouse kidneys at different development stages. An effective solution is to retrain the network model for kidney imaging data of mice of different ages, but this would lead to another problem: manually annotating raw imaging data is very time-consuming and laborious. To circumvent this, we employed a human-in-the-loop approach. As shown in Figure 8a, we first used the pretrained FastCellpose model to segment kidney datasets of mice of different ages. Then, we manually checked and corrected the reconstructed mask. The corrected annotations along with the original imaging data formed the new training data. In this way, it only took 0.5 h to obtain new annotated training data, which was 20 times less than traditional manual annotation from scratch (Figure 8b). We used new training data to perform transfer learning on the pretrained model to obtain optimal networks for the kidneys of mice of different ages.

**Figure 9:**
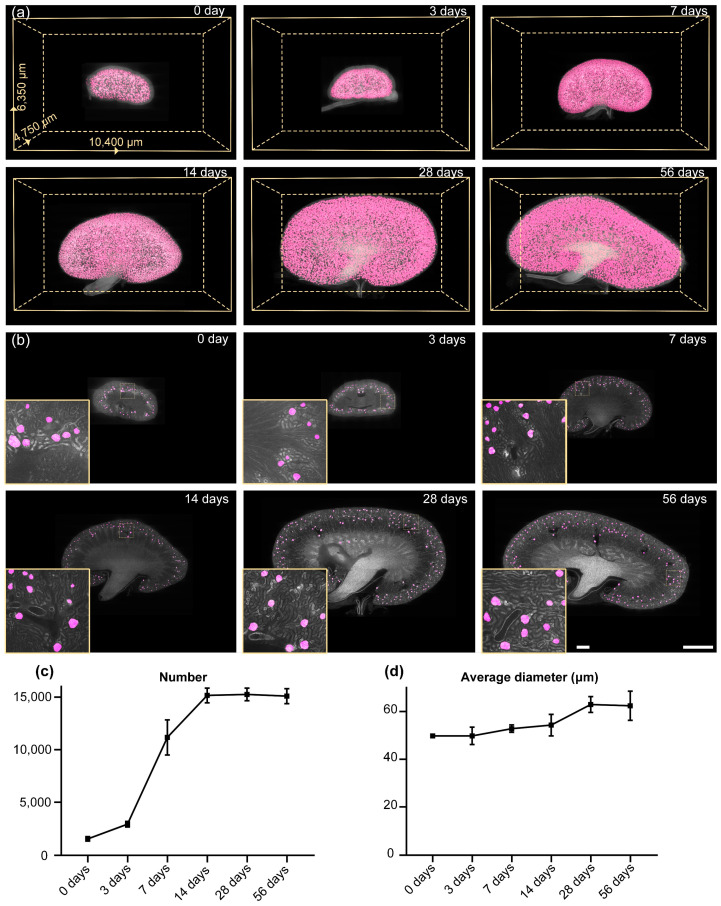
Glomeruli segmentation and analysis results of mouse kidneys of different ages. (**a**) Three-dimensional visualization of the segmentation results of mouse kidneys at different development stages. (**b**) Two-dimensional visualization of the segmentation results of mouse kidneys at different ages. Segmentation results (magenta) are shown overlaid with the original data. Scale bar, 1000 μm; 100 μm for the enlarged image. (**c**,**d**) The development curves of the mouse glomeruli. The number (**c**) and the average diameter (**d**) of glomeruli in mouse kidneys of different ages are exhibited.

Figure 9 demonstrates the segmentation results of our method on mouse kidneys at different development stages. The detailed information for each mouse whole-kidney dataset is shown in Table 4. The 3D reconstruction results intuitively exhibit the overall morphological changes during kidney development (Figure 9a). From the typical coronal sections of the imaging data, it can be clearly seen that the glomeruli in mouse kidneys of different ages can be accurately identified and segmented by our method (Figure 9b), demonstrating the effectiveness of our method for the unbiased analysis of glomerular development. After segmentation, we performed connected component analysis to obtain the number and morphological parameters of glomeruli. We observed that the number of glomeruli increased rapidly from 3 days to 7 days, and after 2 weeks, the number of glomeruli no longer continued to increase (Figure 9c). The number of renal glomeruli in newborn mice is 1545 ± 210, while the total number of glomeruli in a mature mouse kidney reaches 15,095 ± 714. We also quantified the diameter distribution of the glomeruli. We observed that, at each stage of mouse kidney development, the diameter distribution of glomeruli exhibited a Gaussian distribution. In the kidneys of mice aged 0 days to 2 weeks, the vast majority of glomeruli were distributed in the range of 40 to 60 μm, while in the kidneys of mice aged 2 to 8 weeks, most glomeruli were distributed in the range of 60 to 80 μm. We also found that the average size of the glomeruli increased slowly during kidney development. The average glomerular diameter of a kidney of a newborn mouse is 49.8 ± 3.6 μm. After 4 weeks of development, the glomerular size stops increasing at 63.0 ± 3.3 μm (Figure 9d). From these results, it can be deduced that the development of glomeruli in the kidney is basically complete after 4 weeks. These results further demonstrate the effectiveness of FastCellpose for the automatic and accurate analysis of glomeruli, and the obtained quantitative analysis results are expected to promote further study of kidney development.

### 3.4. FastCellpose for Neuronal Soma Segmentation

**Figure 10:**
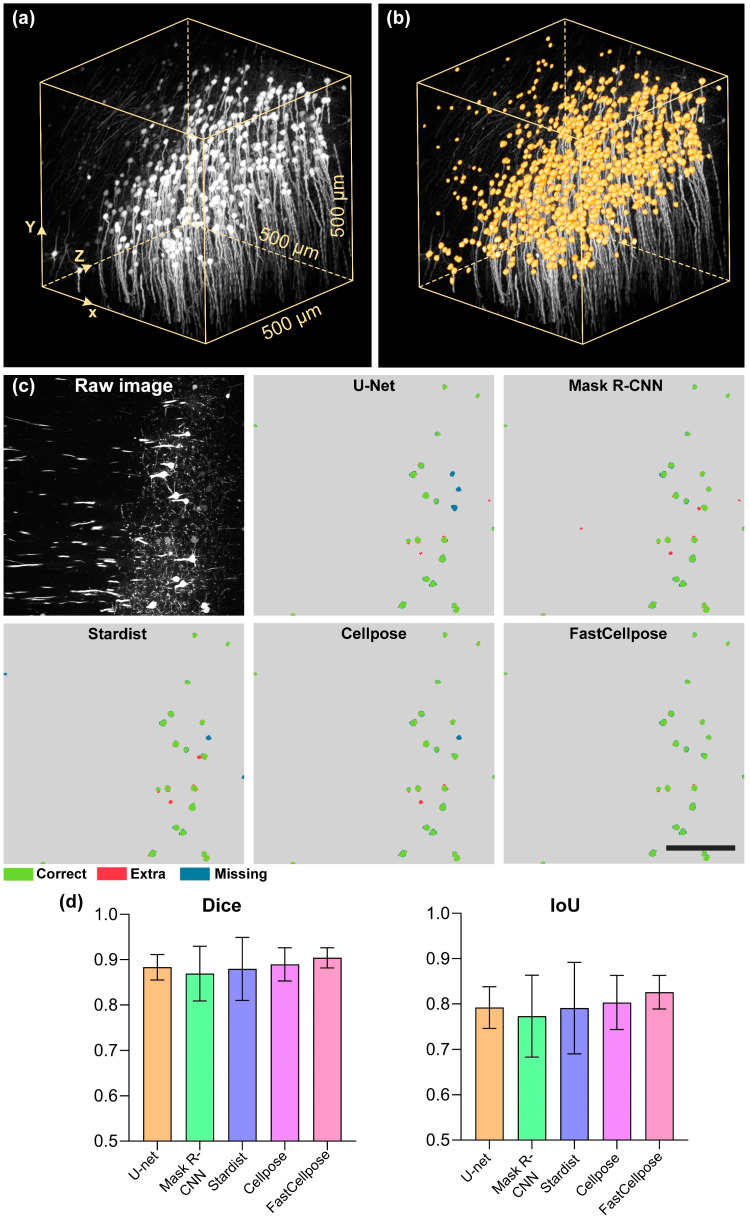
Application of FastCellpose in neuronal soma segmentation. (**a**) Three-dimensional visualization of a typical raw imaging stack from the neocortex in a Thy1-GFP mouse brain. (**b**) Segmentation results of FastCellpose for the imaging data are shown in (**a**). Segmentation results (orange) are shown overlaid with the original data. (**c**) Two-dimensional visualization of the segmentation results of different methods. Scale bar, 150 μm. (**d**) The Dice and IoU scores of the methods are shown in (**c**).

The success of FastCellpose for renal glomeruli segmentation motivated us to further investigate the feasibility of FastCellpose for application in other tissues and organs. We, therefore, tested it on neuronal soma segmentation, which is a crucial step in the quantitative analysis of neuronal morphology [41,42]. A Thy1-GFP mouse brain dataset was selected for testing. The raw data were acquired by a fMOST system at a voxel resolution of 0.32 × 0.32 × 2 μm^3^. We trained the FastCellpose model by using randomly selected coronal sections and the corresponding manually annotated data. After training, a typical image stack (500 × 500 × 500 μm^3^) in the brain cortex was selected to validate the segmentation performance of FastCellpose (Figure 10a).

It can be seen that the neuronal somas were densely distributed in this small area, and there were also interweaving neurites, making accurate segmentation very difficult. Faced with such a complex scenario, our FastCellpose still performed well. The neuronal somas of varying intensities and sizes were accurately identified and segmented, and the interference signals were effectively removed (Figure 10b). We evaluated and compared the performance of FastCellpose with other competitive methods (Figure 10c). Compared with the existing cellular segmentation methods, our method achieved the best segmentation accuracy; the Dice and IoU values were 0.904 ± 0.022 and 0.826 ± 0.037, respectively (Figure 10d). Moreover, our method finished the segmentation of this data block in only 1.7 min, while the original Cellpose model took 20 min, further demonstrating the high efficiency of FastCellpose.

## 4. Discussion and Conclusions

Here, we present FastCellpose for rapid and accurate segmentation of whole-kidney glomeruli. Our framework was based on Cellpose, the most advanced cellular segmentation algorithm at present, and overcame its limitations in processing large-scale data. This was achieved by comprehensive optimization of the network architecture and the mask reconstruction process. Our proposed lightweight network effectively suppressed the risk of overfitting on specific segmentation tasks, achieving improved segmentation accuracy compared to Cellpose and reducing the inference time by sevenfold. Moreover, our optimized reconstruction pipeline significantly accelerated the time-consuming process of gradient tracking, achieving a 16-fold improvement in mask reconstruction speed. These improvements distinguish FastCellpose as a unique segmentation algorithm that can rapidly process large-scale data while maintaining high segmentation accuracy.

Using FastCellpose, we segmented mouse whole-kidney glomeruli and analyzed glomerular changes during the development process. These quantitative results, which were difficult to obtain previously, will effectively promote our understanding of and research into kidney development. Although a series of works based on traditional techniques has been proposed for measuring the glomerular sizes and numbers in whole mouse kidneys [7,8,9], these methods are prone to errors, and the obtained results are not very reliable. For example, the total glomerular count for a healthy adult mouse, derived using MRI and the watershed segmentation algorithm, was only 12,010 ± 447 [9], which deviates significantly from our results. This is mainly because the inherent low resolution of MRI and the low performance of the watershed algorithm led to a large number of glomeruli not being correctly identified. In contrast, our pipeline, which incorporated the high-resolution optical imaging technique and the high-performance deep-learning-based segmentation algorithm, can provide more precise measurements, and the derived results can potentially serve as a benchmark for mouse whole-kidney glomerular count. Therefore, we believe that our approach has broad application potential in kidney research and may facilitate our understanding of the mechanisms of kidney functions and diseases.

Besides whole-kidney glomeruli segmentation, we also demonstrated the effectiveness of FastCellpose for brain neuronal soma segmentation, highlighting the versatility of our method. Although our method is applicable to many types of cells, it should be worth noticing that for cells with elongated morphology, such as neuronal fibers, our method may yield unsatisfactory results. This is because the flow fields for the images of elongated cells may have multiple sinks and result in over-segmentation after gradient tracking. This problem is expected to be mitigated by introducing new intermediate representations such as distance fields.

Overall, the presented FastCellpose framework is a promising approach for the rapid, automated, and accurate analysis of whole-organ datasets. As biomedical research increasingly requires unbiased explorations of whole organisms, we anticipate that our method will be widely employed in many fields.

## Figures and Tables

**Table 1:** Performance comparison of network architectures with different widths and depths.

Segmentation Model	Architecture Parameters			Performance		
	K	N	Trainable Parameters	Dice	IoU	Inference Time
Model 1	32	4	6.60 × 10^6^	0.938 ± 0.018	0.884 ± 0.031	1.50
Model 2	32	2	3.06 × 10^6^	0.942 ± 0.015	0.891 ± 0.026	0.98
Model 3	16	4	1.62 × 10^6^	0.936 ± 0.021	0.881 ± 0.037	0.91
Model 4	32	1	1.49 × 10^6^	0.926 ± 0.017	0.863 ± 0.030	0.84
Model 5	16	2	0.56 × 10^6^	0.947 ± 0.017	0.899 ± 0.031	0.68
Model 6	16	1	0.37 × 10^6^	0.922 ± 0.017	0.856 ± 0.029	0.57

**Table 2:** Segmentation performance under different downsampling scale factor.

Downsampling Scale Factor	Segmentation Performance		Acceleration
	Dice	IoU	
1	0.947 ± 0.017	0.899 ± 0.031	\
2	0.945 ± 0.016	0.894 ± 0.031	3.5-fold
3	0.931 ± 0.015	0.871 ± 0.026	8.0-fold
4	0.896 ± 0.016	0.812 ± 0.020	11.4-fold

**Table 3:** Performance comparison of FastCellpose with other existing methods of glomeruli segmentation.

Segmentation Methods	Trainable Parameters	Performance			
		Dice	IoU	Network Inference Time (s)	Mask Reconstruction Time (s)
U-Net	2.96 × 10^6^	0.913 ± 0.018	0.840 ± 0.031	0.88	\
Mask R-CNN	4.39 × 10^7^	0.888 ± 0.020	0.799 ± 0.033	3.45	\
Stardist	1.43 × 10^6^	0.875 ± 0.022	0.778 ± 0.036	0.81	4.05
Cellpose	6.60 × 10^6^	0.938 ± 0.018	0.884 ± 0.031	1.50	4.68
FastCellpose	0.56 × 10^6^	0.945 ± 0.016	0.894 ± 0.028	0.22	0.28

**Table 4:** Detailed information on the mouse whole-kidney datasets.

Mouse Age (Days)	Number of Datasets	Number of Images per Dataset	Image Size
0	3	736	8497 × 11,712
3	3	852	10,997 × 15,616
7	3	1020	19,993 × 11,712
14	3	1248	22,993 × 17,568
28	3	1527	31,993 × 17,568
56	3	1584	33,996 × 19,520

## Data Availability

The original data and codes of this work can be requested from the corresponding author.
